# Promising future of breast cancer vaccine asking for multidisciplinary collaboration: a literature review

**DOI:** 10.3389/fcell.2025.1578883

**Published:** 2025-04-24

**Authors:** Zhanyi Zhang, Mengyuan Li, Lei Zhang, Maohua Wang, Dequan Liu, Shicong Tang, Yuhua Li, Xuedong Fang, Shengnan Ren

**Affiliations:** ^1^ Department of Plastic and Reconstructive Surgery, The First Hospital of Jilin University, Changchun, China; ^2^ College of Chinese Medicinal Materials, Jilin Agricultural University, Changchun, China; ^3^ State Key Laboratory of Primate Biomedical Research, Yunnan Key Laboratory of Primate Biomedical Research, Institute of Primate Translational Medicine, Kunming University of Science and Technology, Kunming, China; ^4^ Department of Breast Surgery, Peking University Cancer Hospital Yunnan, Yunnan Cancer Hospital, The Third Affiliated Hospital of Kunming Medical University, Kunming, China; ^5^ Department of Gastrointestinal, Colorectal and Anal Surgery, China-Japan Union Hospital of Jilin University, Changchun, China

**Keywords:** cancer vaccine, nanotechnology, biomaterial, drug delivery, multi-omics

## Abstract

Despite improved efficacy of breast cancer vaccine (BCV) made by multidisciplinary collaboration from fields such as materials science and computer science, clinical translation is still far from satisfactory. Herein, we reviewed the evolution trajectory of BCV and summarized the frontier topics and challenges for achieving successful clinical translation. Our analysis suggests that multi-omics and immunoinformatics are increasingly being used to expand target repertoires, and dedicated vaccine platforms are facilitating precise spatiotemporal co-delivery of epitopes and immune modulators. BCV has evolved towards precise delivery, strong immune properties, and combined therapy. Clinical translation requires the joint efforts of experts in clinical oncology, immunology, pharmacology, materials, and computer science.

## Graphical Abstract

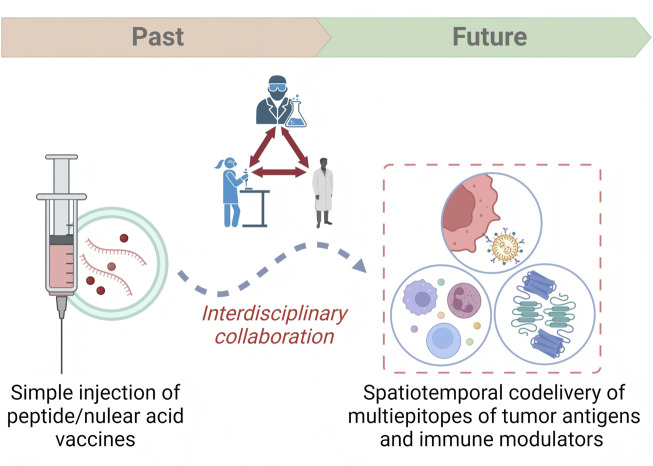

## 1 Introduction

For decades, cancer vaccine has been proposed as an important component of active immunotherapy, aimed at stimulating antigen-specific anti-tumor responses, achieving tumor prevention and regression (Ren et al., 2023; Jeong et al., 2019). Breast cancer is expected to benefit from cancer vaccines for its “weak immunogenicity” (Qian et al., 2023; Vincent et al., 2023; Jiang and Liu, 2023). However, the efficacy remains suboptimal, leading to inconsistent preclinical findings and clinical outcomes (Disis and Cecil, 2022; Jou et al., 2021; Dhanushkumar et al., 2024). The main reason for this clinical dilemma is that the anti-tumor immune response induced by breast cancer vaccines is not strong enough. In general, the causes of weakened antitumor immune responses include: (i) an immunosuppressive tumor microenvironment (TME) that inhibits antitumor immune responses and promotes tumor growth; (ii) a lack of highly immunogenic tumor-associated antigens (TAAs); (iii) insufficient TAA presentation after vaccination; and (iv) immune escape caused by loss of antigen and HLA-class-I molecule expression (Harris et al., 2024; Solinas et al., 2020). Despite this, researchers from various disciplines have never stopped to improve the structure of cancer vaccines, develop new targets, and guiding the continuous evolution of breast cancer vaccines. In this context, a full understanding of the evolutionary process of cancer vaccine development and current research hotspots will help promote the successful clinical translation of breast cancer vaccines by showing the research trends and challenges in this field.

Herein, we briefly summarized the application of DNA vaccines, mRNA vaccines, peptide vaccines, and DC vaccines in the treatment of breast cancer. By studying the evolution of BCV hotpots, and developmental trends, we noticed that the development of BCVs has gone through a serious of evolution and improvement. Compared with the early injection of simple peptides or nucleic acids, co-delivery of multiple epitopes and immunomodulators has been achieved by use of multi-omics and immunoinformatics technologies (Mohsen et al., 2022). The carefully designed vaccine platform not only achieves precise spatiotemporal targeted delivery of the vaccine, but also has the function of modulating the immunosuppressive TME, thereby inducing a strong tumor antigen-specific response (Guo et al., 2022). Data from preclinical studies also signifies the therapeutic value of breast cancer vaccines in combination with other treatments (Zhang et al., 2021). Collectively, it is highly recommended that close multidisciplinary collaboration is the key to solving the dilemma during the clinical translation processes of breast cancer vaccines. This review aimed to provide valuable insights into the present status, possible hotpots, and emerging research trends within this filed, as well as presenting inspiring perspectives.

## 2 Targets and formulation of breast cancer vaccine

### 2.1 Targets of breast cancer vaccine

Vaccine platforms carrying one or more tumor antigens are expected to stimulate specific antitumor immune response. Tumor antigens can be roughly divided into tumor-specific antigens (TSAs) and tumor-associated antigens (TAAs). The ideal target for a breast cancer vaccine should be specifically or highly expressed in cancer cells and be able to elicit T and B cell responses. In breast cancer vaccines, commonly used tumor antigens include HER-2, MUC1, ESR1, CA-125, etc (Dailey et al., 2024). However, it is reported that these vaccines targeting tumor-associated antigens usually induce limited anti-tumor immune effects. With the development of multi-omics, sequencing technology helps to identify more tumor-specific antigens. Vaccine strategies targeting patients’ personalized antigens induce stronger anti-tumor responses and have therefore become a new strategy for developing tumor vaccine targets (Mohsen et al., 2022).

### 2.2 Formulation of breast cancer vaccine

**FIGURE 1:**
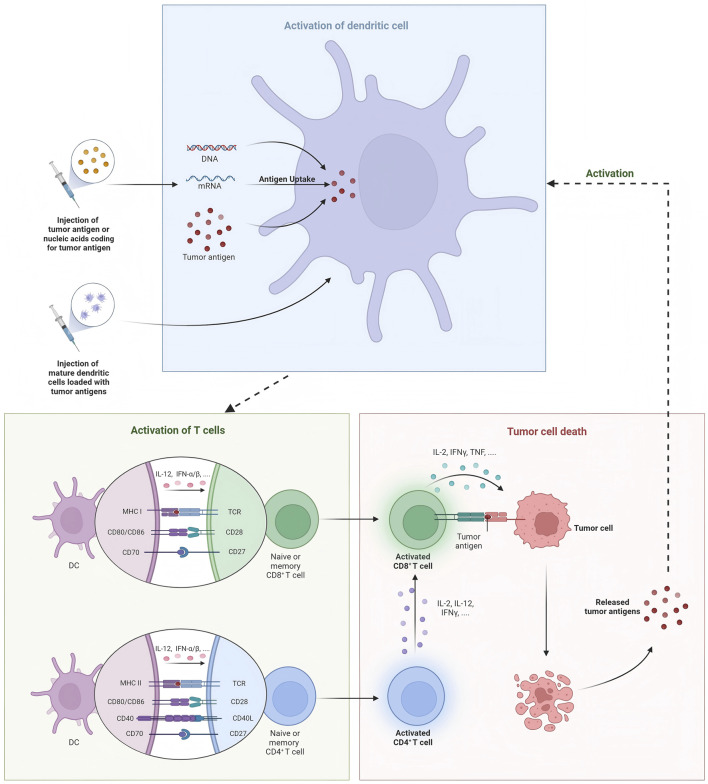
Summarization of therapeutic breast cancer vaccines.

According to the composition of the delivery platform, tumor vaccines include liposomal-based vaccines (Zamani et al., 2019; Zamani et al., 2020; Rastakhiz et al., 2019; Mohammadian Haftcheshmeh et al., 2021; Wallis et al., 2020; Naghibi et al., 2020), virus-like particles (Hu and Steinmetz, 2021; Rolih et al., 2020; Nika et al., 2019), polymeric vaccines (Liu et al., 2020; Xiao et al., 2021; Zhou et al., 2020), and several other nanomaterial-based cancer vaccines. Generally, we often divide cancer vaccines into DNA vaccines, mRNA vaccines, protein vaccines, and DC vaccines. Additionally, preventative vaccine is also illustrated in this section (Figure 1).

#### 2.2.1 DNA vaccine

As nucleic acid vaccines are flexible in antigen selection and are relatively fast, cheap and efficient to produce, DNA vaccines are widely recognized to induce precise and effective immune responses (Brito et al., 2023). HER-2 is one of the most common targets of DNA vaccine to control breast cancer. The importance of DNA vaccines targeting the intracellular segment of ERBB2 in controlling recurrence was demonstrated in a phase I trial after 10-year`s follow-up, which induced specific T cell response after intradermal injection with a remarkable safety profile. Moreover, it reported that increased residual DNA dose at the vaccination site was associated with lower levels of specific response, laying foundation for the next phase II randomized clinical trial (Disis et al., 2023). Since ESR1 mutation is one of the main causes for endocrine therapy resistance, Dailey et al. designed an adenoviral ESR1 vaccine encoding ESR1 mutation forms and observed that vaccination effectively prevented the clinical emergence of *ESR1*mut + BC and the development of *ESR1*mut-mediated endocrine therapy resistance (Dailey et al., 2024).

The rapid development of nanotechnology and bioinformatics has greatly contributed to improvement and optimization of DNA vaccines, which not only provides more epitope information, but also enables simultaneous delivery of multiple components and induces a stronger immune response. Recently, immunoinfomatics servers were applied to analyze the immunogenic properties of cancer testis antigen BORIS, including CTL epitopes and B cell epitopes, and proper linkers incorporating Th epitope and TLR-4/MD-2 agonist to design a novel multi-epitope DNA vaccine (Mahdevar et al., 2022). Molecular dynamic (MD) and other analysis methods were then used to calculate the immunogenicity, stability, intracellular distribution and function of the vaccine. In addition, post-translational modification, an important factor affecting the effect of DNA vaccines, was taken into account, and the optimal multi-epitope DNA vaccine was ultimately obtained through computer simulation. Besides, it is well established that potent immune responses can be induced when cancer vaccines are combined with other treatments. Lately, multi-epitope DNA vaccine was developed with sequencing and *in silico* neoantigen prediction pipelines. When combined with anti-PD-L1 antibody, the DNA vaccine induced robust antitumor response in preclinical models (Li et al., 2021)Moreover, nanotechnology has also contributed a lot to designing multicomponent and multivalent cancer vaccines, and we refer readers to these reviews (Li et al., 2022; Lim et al., 2020).

#### 2.2.2 mRNA vaccine

Since the widespread use of COVID-19 mRNA vaccines, the development of mRNA vaccines to control tumors has received increasing attention. Currently, mRNA vaccines have been widely studied in the field of breast cancer. We recommend some excellent reviews in this field to readers (Li et al., 2023; Haque et al., 2021). Immunoinformatics has now been recognized as a powerful assistance to mRNA vaccine development. Lu et al. developed self-adjuvant mRNA vaccine targeting multi-epitopes of CA-125 with CD40 L and MHC-I targeting domain, and simulated the immune responses using C-ImmSim algorithm (Lu et al., 2023). It was estimated that cross-presentation by DCs to Th1 cells was remarkably higher than to Th2 or Th17 cells, and induced the production of IFN-γ and CTL response. This strategy was characterized by being scalable and can be used as a cost-effective approach to design breast cancer vaccines.

In terms of instability and easy degradation of RNA vaccines during delivery, nanotechnology is expected to solved the problem, such as liposome-mediated vaccine delivery system. Guo et al. has developed a novel vaccine delivery platform composed of α-Galactose ceramide/cationic liposome complex (α-GC-Lip) and peptide-encoding mRNA (Guo et al., 2022). In addition to delivery neoantigen to antigen-presenting cells (APCs), the vaccine also promoted DCs maturation via upregulation of bone marrow-derived cells (BMDCs) surface molecules and cytokine secretion. In mouse models, vaccination combined with anti-PD-1 therapy not only induced antigen-specific T cell responses, but also activated innate immune responses and reshaped the inhibitory TME, achieving robust anti-tumor effects. mRNA vaccine can also be used in combination with anti-CTLA-4 therapy. mRNA vaccine encoding MUC1 was encapsulated in liposome to enhance delivery efficacy. Subcutaneous injection was administrated to mice model in combined with anti-CTLA-4 therapy, and significant inhibition of tumor growth in mice was observed (Liu et al., 2018). In addition, it is also possible to reshape the TME while delivering mRNA vaccine via regulating the expression level of inhibitory or stimulatory genes, increasing the number of immune-infiltrating cells, and finally improving the effect of immunotherapy (Zhang et al., 2021).

#### 2.2.3 Peptide vaccine

Generally speaking, peptide vaccines contain 20–30 amino acids, which can induce T cell and B cell responses and exert synergistic effects in combination with other therapies. Recently, in a phase I trial (NCT02364492), seven breast cancer patients were immunized with the fully synthetic glycopeptide cancer vaccine MAG-Tn3, which induced Tn-specific humoral responses with antitumor capabilities (Rosenbaum et al., 2020). In an early phase II study, a cocktail of 19 peptide vaccines targeting 11 different tumor-associated antigens (TAAs) was evaluated as monotherapy in 14 patients with metastatic breast cancer (UMIN000014616) (Toh et al., 2020). After vaccination, specific antibody and cell immune response were detectable and associated with overall survival. It has also revealed that low baseline C-reactive protein and chemotherapy history are risk factors for poor benefit from vaccine treatment.

Mohsen et al. used immunoinformatics to develop an optimized peptide vaccine using whole exome sequencing and mass spectrometry-based immunopeptidomics to identify tumor neoantigens (Mohsen et al., 2022). Four neoantigen peptides packaged with TLR-9 ligands were coupled together by click chemistry for *in vivo* co-delivery. Vaccination of mice resulted in neoantigen-specific CD4^+^ and CD8^+^ T cell responses and TME repolarization, representing a feasible and practical bedside personalized treatment approach. Development of nanotechnology has enabled the design of versatile vaccine delivery platforms, such as multi-epitope vaccine, stimuli-responsive vaccine and injectable hydrogel vaccine. It has been reported that liposomal vaccines can carry both Th and cytotoxic T lymphocyte epitopes and activate both CD4^+^ and CD8^+^ T cells, producing specific anti-tumor immune response in mice models with therapeutic and preventive effects (Zamani et al., 2019; Zamani et al., 2020). To achieve the co-delivery of neoantigens and more effective adjuvants, Su et al. reported polymeric nano vaccines loading model antigen and STING agonist cGAMP, which disassembled in acidic conditions (Su et al., 2022). The vaccine component reshaped the suppressive tumor microenvironment through activation of STING pathway, and improved the immunotherapy effect when used in combination with ICB. The temporal and spatial delivery of nanomedicine and nano vaccine was achieved by loading curcumin polymer and tumor antigen peptide into thermosensitive nanoparticles (Liu et al., 2020). This nano vaccine was injected into the surgical site and triggered a strong T cell response, thus effectively inhibiting local recurrence and lung metastasis. In addition, Chen et al. constructed a thermosensitive hydrogel-encapsulated peptide vaccine and implanted it into the surgical site, which produced a sustained immune response in mice with orthotopic breast cancer (Chen et al., 2022). This implantable nano vaccine has unique advantages in co-delivery and large-scale production, and effectively controlled cancer recurrence and distant metastasis.

#### 2.2.4 DC vaccine

DC vaccines induce DC activation *in vitro* and then infuse them into the body, activating T cell responses by presenting tumor antigens and generating specific anti-tumor immunity. Two important measures in developing DC vaccines are the selection of appropriate DC subtypes and the induction of mature states. Current studies have found that cDC1 vaccines have achieved remarkable results in both preclinical and clinical studies. In DC vaccine, HER-2 is the most commonly used target. In a clinical study, researchers evaluated cDC1 vaccine pulsed with HER-2 peptide in early invasive breast cancer (IBC) and ductal carcinoma *in situ* (DCIS) (Lowenfeld et al., 2017). After vaccination, anti-HER-2 CD4 immune response was detected in blood and sentinel lymph nodes of breast cancer patients, and patients with DCIS exhibited much higher rate of pathologic complete response (pCR) than IBC patients (28.6% vs. 8.3%). Besides, a clinical study used six peptides targeting HER-2 to activate DC1 *in vitro* and induced a long-lasting Th1 response in 27 patients with DCIS who overexpressed HER-2 (Koski et al., 2012a). Subsequently, this team conducted a serious of phase I and II studies to determine the efficacy of DC vaccines targeting HER-2 in patients with DCIS and different subtypes of BC (Sharma et al., 2012; Datta et al., 2015). Lately, Ramamoorthi et al. reported that intratumoral delivery of HER-2 targeted DC vaccine combined with anti-HER-2 therapy elicited robust antitumor response in mice models (Ramamoorthi et al., 2022). Compared with anti-HER-2 therapy and chemotherapy, DC vaccines therapy generated stronger CD4^+^ and CD8^+^ T cell response and played a synergistic role by enhancing antibody-dependent cellular cytotoxicity (ADCC) of anti-HER-2 treatment. In addition to HER-2, HER3 is also a common target for DC vaccines. Using peptide screening methodology, nine HER3 peptides with capability to binding MHC-II were identified, and the HER3 peptide-pulsed DC vaccine generated specific CD4^+^ Th1 antitumor immune response impeding tumor growth in HER3-experssing mouse tumor models. Moreover, inspired by Sipuleucel-T therapy for prostate cancer, Munoz et al. developed a vaccine platform consisting of autologous DCs pulsed with tumor membrane vesicles (TMVs) encapsulating personalized tumor antigens, which showed anti-tumor effects in HER2-positive breast cancer and triple-negative breast cancer (Munoz et al., 2021). Several clinical trials have investigated the safety and efficacy of DC vaccines in the treatment of locally advanced or metastatic breast cancer, and we refer readers to these reviews for additional information (Qian et al., 2023; Gautam et al., 2022; Gautam et al., 2024). DC vaccines combined with other types of cancer vaccines also show great therapeutic potential. Berneman et al. conducted a single-arm phase I/II clinical study testing mRNA/DC vaccine in 12 patients with metastatic breast cancer and achieved median overall survival (OS) of 41.9 months, and this survival benefit was mainly associated with antigen-specific type 1 CD4^+^ and/or CD8^+^ T-lymphocyte responses (Berneman et al., 2025). Lapenta et al. developed a personalized DC vaccine using oxidized tumor cell lysate, which retained tumor antigen characteristics in a 3D organoid culture environment and induced effective Th1 immune responses and strong cytotoxic activity (Lapenta et al., 2024). In addition, by whole exome and RNA sequencing, the researchers synthesized HLA class II-restricted long peptides containing epitopes with high affinity for HLA class I and prepared DC vaccines. Subsequently, specific T cell clones were generated in two of the three vaccinated patients, and no relapse was observed in all three patients (Morisaki et al., 2024).

Compared with DC vaccine pulsed *in vitro*, *in situ* DC vaccines are developed by use of nanotechnology. Huang et al. applied engineered exosomes expressing α-lactalbumin (α-LA) loaded with immunogenic cell death (ICD) inducers ELANE, and TLR3 agonist Hiltonl (Huang et al., 2022). By utilizing the specific homing properties of exosomes, HELA-Exos were successfully accumulated in the TME, inducing tumor ICD to release tumor antigens, and observed that *in situ* cDC1s were activated and initiated tumor-reactive CD8+T cell responses, showing strong anti-tumor activity in mouse models and human breast cancer organoids. This *in situ* DC vaccine shows advantages different from traditional DC vaccines and has important potential for wide application. Moreover, Wang et al. reported a novel *in situ* DC vaccine integrating multiple therapeutic strategies, which realized photothermal therapy (PTT) and chemodynamic therapy (CDT) and immune modulation of breast cancer (Wang et al., 2024). They designed an “*in situ* nano vaccine” Au/CuNDs-R848 that combined dual-mode imaging guidance capabilities and PTT/CDT synergistic therapy. In the tumor site, this *in situ* DC vaccine released tumor antigens to induce DC maturation and activation, also enhanced tumor immunogenicity and reversed the inhibitory TME through multiple mechanisms, thereby enhancing the therapeutic effect on primary and metastatic breast cancer.

#### 2.2.5 Microorganisms-based vaccine

The use of viral or bacterial vectors to deliver TSAs or TAAs is another common vaccine strategy. Viral or bacterial vectors can not only act as adjuvants to increase antitumor immune responses, but the natural ability of viruses to infect human cells can also be used for antigen delivery to improve delivery efficiency (Gebre et al., 2021; Asad et al., 2017; Shanmugaraj et al., 2020). In addition, the oncolytic ability of some viruses can also be modified to selectively attack tumor cells. For example, oncolytic virotherapy works by inducing targeted destruction of tumor cells and stimulating immune responses against viral and tumor cell antigens, thereby generating long-term immune memory. Forčić et al. designed an intratumoral oncolytic virotherapy as a neoadjuvant treatment in preparation for successful surgery. Forty-five months after surgery, no signs of recurrence was observed (Forcic et al., 2024). Other viral vaccines, such as recombinant poxvirus vaccines and recombinant fowlpox virus vector vaccines, can also provide clinical benefits for patients with metastatic breast cancer (Mohebtash et al., 2011; Heery et al., 2015). Huang et al. designed a novel cancer vaccine (meAAV) in which adeno-associated viruses were optimized to include tumor neoantigens, TLR9 inhibitory fragments, PD-1 traps, and PD-L1 miRNA (Huang et al., 2023). In tumor-bearing mice, an increase in neoantigen-specific T cell responses was observed when radiation therapy was combined with RT. Cancer vaccines using bacteria as vectors also have many advantages. Certain bacterial strains, such as *Clostridium*, are highly active in the anaerobic/anoxic TME, and some auxotrophic strains, such as *Salmonella*, may be attracted to the metabolic features in the TME (Dang et al., 2001; Zhao et al., 2005). Shahabi et al. developed a highly attenuated *Listeria* vector, LmddA. This construct proved to be effective in overcoming immune tolerance to the HER2/neu autoantigen (Shahabi et al., 2011). However, there are still several challenges in the clinical translation of microbial vaccines. Improving the clinical safety of recombinant vaccines could facilitate their acceptance in therapeutic applications.

#### 2.2.6 Preventative breast cancer vaccine

The application of preventative breast cancer vaccines aims to block the carcinogenesis process at precancerous stage such as atypical hyperplasia or lobular carcinoma *in situ*. This strategy includes the use of a variety of vaccines targeting the above tissue-specific or tumor-specific antigens or proto-oncogenes, such as DNA vaccines and DC vaccines. In a previous study, α-LA was chosen as the target of prophylactic cancer vaccine since it is only expressed in lactating mammary epithelial tissues and induced specific immune response, showing preventative and therapeutic effect in autochthonous breast cancer in MMTV-PyVT transgenic mice (Jaini et al., 2010). Currently, many clinical trials have used tumor vaccines targeting HER-2 to prevent breast cancer (Zachariah et al., 2021). In the FVB-huHER2 transgenic mouse model, prophylactic HER2 vaccination significantly prevented tumor formation in mice, while in the xenograft tumor model, injection of serum from DNA-vaccinated mice significantly inhibited the growth of human HER2-positive tumors (De Giovanni et al., 2014). Additionally, researchers also developed preventive vaccine that simultaneously targets IGFBP-2, IGF-IR, and HER-2 proteins. The effect of preventing breast cancer progression was observed in 65% of transgenic mice. Based on this, a Phase I clinical trial is also underway to observe whether this multi-antigen vaccine produces reliable immunity (Disis et al., 2013). Moreover, Nordin et al. integrated the cytotoxic T cell (GP2) and B cell (P4) peptide epitopes of HER2/neu to prepare a peptide vaccine and observed that compared with vaccination with GP2, a stronger preventive and therapeutic effect against HER2-positive breast cancer was achieved due to the production of neutralizing antibodies (Nordin et al., 2021).

In addition to reduce the incidence of breast cancer, preventive vaccines are also used to prevent breast cancer recurrence and metastasis, which represents an important aspect of the clinical application of tumor vaccines. Peptide vaccines targeting HER-2 have achieved considerable results in preventing breast cancer metastasis (McCarthy et al., 2021). AE37 vaccine is a hybrid polypeptide vaccine, including AE36 (the intracellular part of HER-2), li-key (the core part of MHC-II class molecule), and GM-CSF as adjuvant. It has been used in several clinical studies of breast cancer and prostate cancer because it contains li-key that can enhance immune response and have epitope amplification effect, thereby inducing CD4^+^ T cell response targeting HER-2. TOP2A was also selected as target for preventative BC vaccine. Using computer analysis technology, the antigenic epitope of TOP2A was screened out through multiple algorithms, and a peptide vaccine was prepared with CpG as an adjuvant, demonstrating favorable effects against triple-negative breast cancer (TNBC) (Lee et al., 2023). In the mouse model, through pre-immunization and injection of tumor cells, it was observed that the tumor incidence and growth rate were affected after transplantation, and vaccine-specific T cell responses were detected. In a retrospective study by Zelba et al., a prophylactic peptide vaccine was administered intradermally in four BC patients (1 case of TNBC, 2 case of ER positive BC, and 1 case of HER2 positive BC) who had achieved complete remission after chemotherapy, followed by subcutaneous and adjacent adjuvant administration (Zelba et al., 2022). Personalized neoantigens were identified using somatic tumor mutation analysis and patients received vaccination consisted of HLA-I and -II peptides. Durable CD8^+^ and CD4^+^ T cell responses against HER-2 were developed in all patients several months after vaccination. Although preventive effects have been achieved in TNBC and HER2 positive BC, vaccines targeting hormone receptor positive subtypes of breast cancer have not yet been reported, which also emphasizes the importance of a comprehensive understanding of breast carcinogenesis and cancer immune modulation. As for the challenges of preventive breast cancer vaccines, the main problems are the generation of long-lasting antigen-specific immune responses and biosafety issues, which require more rationally designed clinical studies and precise and reliable pharmacological studies before clinical application.

## 3 Challenges and perspectives

**FIGURE 2:**
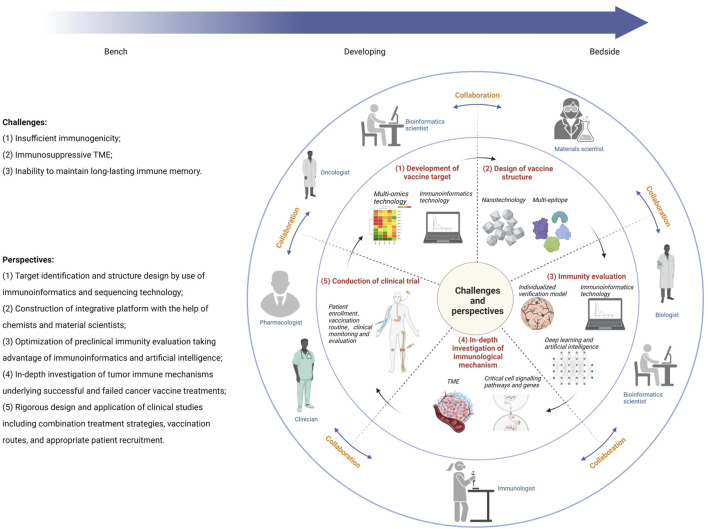
Illustration of multidisciplinary collaboration to overcome challenges in breast cancer vaccine development.

Generally, a tumor vaccine needs to go through these processes from bench to bedside, namely, establishment of antigenic targets (identification, immunogenicity evaluation, antigenic epitopes prediction, and production of tumor antigens), formulation structure design (selection of adjuvants, linkers, and delivery platforms), immunity evaluation (*in vitro* and *in vivo* models to evaluate the immune responses of vaccines), and clinical application (design and implementation of clinical trials). Bibliometric analysis shows that BCV has evolved from simple nucleic acid and protein injections and has undergone a series of changes (see Supplementary Information). Therefore, the challenges faced by BCV development and perspectives for accelerating clinical translation are introduced in the following aspects (Figure 2).

### 3.1 Development of vaccine target

This is the key to design tumor vaccines with potent anti-tumor effect. With the development of multi-omics technology and single-cell sequencing technology, the identification of tumor neoantigens has become increasingly successful. In addition to traditional coding genes, it has recently been reported that circRNAs encode cryptic peptides with immunogenicity that trigger T cell responses (Huang et al., 2024). Noteworthily, heterogeneity is still a key factor affecting the efficacy of immunotherapy, especially tumor vaccines. At the same time, tumor cells are constantly evolving and adjusting their antigen expression profiles during treatment, and creating an immunosuppressive microenvironment to escape the attack of the immune system. Therefore, using multi-omics technology and immunoinformatics to dissect tumors will enable real-time and accurate understanding of the expression profile of tumors, thereby finding the best targets and improving the efficacy of tumor vaccines (Dhanushkumar et al., 2024). Aparicio et al. identified a total of 98 HLA-I class neoantigens for eight patients using various sequencing and algorithms, and then validated the immunogenicity in transgenic mice (Aparicio et al., 2022). Besides, computer tools can be used to predict epitopes of antigen, including TAP epitope and B cell epitope. Prediction of antigenic epitopes will facilitate vaccine structure optimization and enhance the potential to stimulate potent and specific immune responses. For instance, an online tool is developed for analyzing antigenicity, VaxiJen, which predicts the physicochemical properties of protein and provides appropriate vaccine targets and immunotherapy routes for researchers to select (Schroeder and Aebischer, 2011).

### 3.2 Design of vaccine structure

After identifying and selecting reliable tumor antigens, effective delivery to target cells and further processing and presentation to activate efficient anti-tumor immune response is an equally critical process. It is reported that improved immune efficacy is observed in multiple-epitope vaccines than in mono-epitope vaccine (Garzia et al., 2024). Current vaccine formulations include antigen epitope polypeptides or gene sequences encoding epitopes, linkers, immune adjuvants, and delivery platforms. On the one hand, rational design of antigen epitope structure is crucial for the binding of antigen to immune receptor. Currently, there are a variety of computer analysis methods and tools that can assist in the structural prediction of antigen epitopes, and conduct comprehensive analysis and scoring of the number of applied epitopes to select the best combination. However, the largest number of antigen epitopes do not guarantee the most effective immune response, because secondary and tertiary structures also have an important impact on the immune performance of vaccine. Molecular dynamics (MD) simulation and docking analysis are widely used to provide valuable and comprehensive data on the immune binding ability of vaccines in advance, making it easier for researchers to optimize vaccine structure for maximum efficacy (Cole et al., 2008). On the other hand, the selection of appropriate linkers and adjuvants is equally important for successfully activating immune system. Adjuvants have the functions of reducing antigen release, enhancing antigen uptake and presentation by APCs, and promoting proliferation of dendritic cells and macrophages. They are widely believed to generate strong antigen-specific immune responses to BC vaccines. We refer readers to these excellent reviews for detailed information on cancer vaccine adjuvants (Zhang X. et al., 2023; Zhao et al., 2023; Ben-Akiva et al., 2025). Common linkers and adjuvants used in current vaccine development are already well known, but the application of immunoinformatics, especially MD, can help researchers select appropriate adjuvants to obtain a long-lasting and effective immune response (Yang and Yu, 2009).

As aforementioned, nanotechnology-based delivery platforms have been widely used in developing tumor vaccines, which can achieve reliable and targeted delivery of vaccines components. Nano vaccines, taking advantage of the diversity of platforms, are able to simultaneously deliver multiple antigen epitopes, adjuvants, and immunomodulators to induce potent and durable immune response. Nano vaccine has played an important role in development of tumor vaccines, and related research has exploded in recent years. Compared with conventional tumor vaccines that directly delivered nucleic acids or peptides, the application of nanotechnology has successfully promoted the evolution and improvement of tumor vaccines, and has superior performance in improving the richness of cargo, efficacy of targeted delivery, biological stability and compatibility, and generating lasting immune responses. Specifically, liposomes can be easily integrated into native cell membranes, where they serve as anchors for modified motifs or exert their intrinsic functions (i.e., stimuli-responsiveness). As for polymeric nanoparticles, polymers can be strategically selected/designed based on their physical and chemical properties to modulate appropriate immune responses for specific breast cancer subtypes. Regarding the application of nano vaccines, we recommend readers to refer to these excellent review articles (Ahmad et al., 2022; Davodabadi et al., 2022).

Despite the success in preclinical and clinical studies, there are still some challenges and obstacles that need to be addressed. First, in order to advance the nanoplatform to the clinic, it is crucial to develop innovative methods for nanocarrier formulation that are more effective, safer, and simpler. Second, the lack of quality control limits the large-scale industrial production of nanovaccines. Therefore, the integration of advanced technologies is of great significance to minimize side effects and toxicity. For example, toxic molecules can be knocked out or downregulated with the help of genetic engineering. Proteomic and metabolomic analysis can help characterize the molecular composition and metabolites of natural ingredients. Artificial intelligence can help simulate the interactions between nanocarriers and biological fluids as well as cell membranes.

### 3.3 Immunity evaluation

Initially, mouse tumor models were commonly used to verify the effectiveness of vaccine regimens in activating immune responses. In recent years, personalized cancer vaccines are developed by use of multi-omics sequencing technology and single-cell sequencing technology, which requires more sophisticated models to evaluate the immunity, and patient-derived tumor organoids have become one of the solutions. However, these methods have certain limitations. Mouse orthotopic tumor models cannot replicate tumor heterogeneity and have a relatively simple immune system. Organoid models are time-consuming and costly. They cannot truly reproduce the complex and delicate tumor immune microenvironment in human body. Therefore, reliable and relatively simple tumor assessment models need to be developed with high fidelity. Firstly, immunoinformatics technology can describe the immune system in advance through immune simulation to evaluate the efficacy of vaccines, and design personalized vaccines based on the characteristics of patient’s immune system. Xu et al. developed an adaptive immunogenicity prediction model that can quickly identify tumor-associated endogenous antigens through a meta-learning strategy. After transfer learning of peptide-HLA (pHLA) elution ligand data, the model also showed relatively good performance in revealing immunotherapy mechanisms (Xu et al., 2024). Khanam and colleagues analyzed and designed a multi-epitope vaccine based on WT1 and NY-ESO-1 antigen proteins through immunoinformatics technology, which can stimulate strong humoral and cellular immune responses in the target organism (Khanam et al., 2024). Deep learning and artificial intelligence (AI) are also used to predict immune escape mechanisms, optimize dosages and dosing regimens, and help design subsequent clinical trials. For example, C-IMMSIM has been used to design and evaluate vaccine effects in TNBC (Dominguez et al., 2003). Yang et al. developed a deep learning framework HLAIImaster based on the attention mechanism. The model has significantly improved the forward prediction ability over existing tools (Yang et al., 2024). Secondly, the use of humanized mice to establish patient-derived xenograft (PDX) models can provide a research model that is closer to the real internal environment of the human body, but it is costly and time-consuming. With the advancement and development of materials science, 3D bioprinting technology can quickly establish a multi-cell symbiosis system, while having advantages of high throughput and low cost. It has a certain degree of fidelity in simulating the complex immune environment in body, and is a reliable model that can be applied to tumor vaccine research, which is convenient for clinical development (Zhang Z. et al., 2024). Using 3D bioprinting technology, Kim and coworkers developed a cancer-on-a-chip to mimic the TME and predict effects of immunotherapeutic agents (Choi et al., 2023).

### 3.4 In-depth investigation of immunological mechanism

The dynamics and heterogeneity of immunosuppressive mechanisms in TME are major reasons for the failure of tumor vaccines and other immunotherapy strategies. Therefore, in-depth research on the altered molecular mechanisms and metabolic pathways related to T cell exhaustion and dysfunction in TME, exploring solutions to improve APC processing and presentation of tumor antigens, and clarifying the regulatory effects of inhibitory components on immune responses will help to gain a deeper understanding of the success or failure of tumor vaccines and other immunotherapies, thereby promoting the continuous optimization and improvement of tumor vaccines. Recently, it was observed that phenotypic changes in tumor-associated macrophages (TAMs) were responsible for the immunosuppressive mechanism of BRCA-mutated breast cancer, and STING agonists could reverse these changes and enhance anti-tumor effects (Wang et al., 2022). Vaccine platform can also deliver some immune modulatory components, such as cGAMP, LAG3-Ig, and miRNA, etc., which will play a role in regulating and reshaping the inhibitory immune microenvironment (Malla et al., 2024). In terms of the macro immune environment, cancer occurrence and progression have an important regulatory effect on bone marrow progenitor cells and bone marrow function, causing bone marrow dysregulation, weakening local and systemic anti-tumor immunity, and ultimately shaping the tumor-promoting TME. Tumor angiogenesis plays an important role in the formation of an immunosuppressive TME and promotes the resistance of breast cancer cells to immunotherapy drugs (Mou et al., 2024). In addition, a multi-omics study of HER2+ breast cancer found that tumor cells can evade anti-tumor immunity by mimicking the anti-inflammatory mechanism of the central nervous system (Li et al., 2024). These complexities indicate that the design of effective immune-based therapies will require a comprehensive understanding of molecular basis of the interaction between local and systemic immunity.

### 3.5 Conduction of clinical trial

Currently, a variety of breast cancer vaccines targeting HER2 and other personalized antigens have been applied in clinical trials to evaluate their therapeutic value (Table 1). Although most tumor vaccines induce strong immune responses in preclinical studies, they often fail to produce effective and durable anti-tumor effects in the clinical research stage. Therefore, a rational and reliable research design should carefully screen the appropriate subject population before the trial begins, conduct certain assessments and predictions of patients before treatment begins, and monitor and adjust the plan at any time during the application process. At present, AI has been utilized by some companies to assist in the design of clinical research plans, and saved a lot of time-consuming and costly work by analyzing patient records to evaluate the feasibility of the experiment (Nieuwkoop et al., 2019). AI can also effectively assist in the staging, diagnosis, and prognosis prediction of breast cancer, and provide important support for the design and evaluation of clinical trials (Zhang J. et al., 2023). The establishment of a clinical evaluation system for immunotherapy is also crucial to the successful application of tumor vaccines. Before clinical application, candidate patients are screened through reliable targets and strategies, and the secretome and clinical characteristics of systemic cancer patients are evaluated. Combined with the understanding of tumor somatic cell evolution, it can provide a basis for the development of a treatment framework to guide patient immune stratification and immunotherapy decisions. During the application process, identifying and detecting relevant indicators of immune memory can help to evaluate the treatment effect in a timely and accurate manner. At the same time, reflection and in-depth analysis of treatment cases can also help to accurately select the appropriate population for tumor vaccines and improve treatment effects.

**TABLE 1:** Information of breast cancer vaccines applied in clinical trials.

Trial phase	Vaccine formulation	Targeted tumor antigen	Adjuvants	Breast cancer subtype	Primary objectives	Outcomes	Trial registration	Ref
Phase I	DNA vaccine	ERBB2 intracellular domain	Soluble granulocyte-macrophage colony-stimulating factor	Advanced-stage ERBB2-positive breast cancer	Safety, immunogenicity	The majority of vaccine-related toxic effects were grade 1 and 2; central memory T cells were significantly increased in optimally vaccinated patients	NCT00436254	Disis et al., 2023
Phase I	Peptide vaccine	Glycopeptide MAG-Tn3	AS15 immunostimulant	Localized breast cancer at high-risk of relapse	Safety, immunogenicity	All vaccinated patients developed high levels of Tn-specific antibodies	NCT02364492	Rosenbaum et al. (2020)
Phase II	Peptide vaccine	Mixed 19‐peptide	Incomplete Freund’s adjuvant (Montanide ISA-51VG)	Advanced metastatic triple‐negative breast cancer	Safety, immunogenicity	No severe adverse events related to the vaccination were observed; the median OS was 11.5 or 24.4 months	UMIN000014616	Toh et al. (2020)
Phase I	DC vaccine	HER2	-	Ductal carcinoma *in situ* (DCIS), early invasive breast cancer (IBC)	Safety, clinical responses	Vaccination by all injection routes was well tolerated; the pathologic complete response in DCIS was 28.6%, the pathologic complete response in IBC was 8.3%	NCT02061332	Lowenfeld et al. (2017)
Phase I	DC vaccine	HER-2/neu	Clinical-grade bacterial endotoxin (LPS)	Ductal carcinoma *in situ*	Safety, immunogenicity	Immune responses were observed up to 52 months post-immunization	-	Koski et al. (2012b)
Phase I	DC vaccine	HER-2/neu	NIH reference standard LPS	Ductal carcinoma *in situ*	Safety, feasibility	Only grade 1 and 2 toxicities were observed; vaccines induced decline and or eradication of HER-2/neu expression	NCT001070211	Sharma et al. (2012)
Phase I	DC vaccine	HER2	NIH reference standard LPS	HER2^pos^-invasive breast cancer	Immunogenicity	Anti-HER2 CD4^+^ Th1 response was induced	NCT02061423	Datta et al. (2015)
Phase I/II	DC vaccine	HER2	Granulocyte-macrophage colony-stimulating factor	Metastatic breast cancer	Safety, immunogenicity	Treatment was well tolerated; 46% of patients had stable disease after therapy	NCT0088985; NCT00266110	Vincent et al. (2023)
Phase I/II	DC vaccine	WT1	-	Metastatic breast cancer	Safety, immunogenicity, and clinical activity	Vaccination was well tolerated; The disease control rate and overall response rate were 74.4% and 12.8%, respectively	NCT01291420	Berneman et al. (2025)
Phase I	Preventative vaccine	Neoantigens	-	Triple-negative breast cancer	Safety, immunogenicity	The vaccinations were well tolerated; recurrence-free survival was 87.5% at a median follow-up of 36 months	NCT02348320	Zhang et al. (2024b)

## 4 Conclusion

Since the concept of tumor vaccines was proposed, researchers have never ceased to develop and evolve the performance of tumor vaccines, although their clinical efficacies have been limited. As advances in different research fields have been successfully applied to the research and development of tumor vaccines, tumor vaccines have evolved from the initial simple injection of nucleic acids or proteins to construction and production of personalized vaccines for patients. At the same time, with the help of nanotechnology and immunoinformatics tools, as well as the combined application of immunotherapy strategies such as ICB, multi-epitope and multi-component tumor vaccines are continuously developed demonstrating strong immune response efficacy *in vivo* and *in vitro* experiments, which can achieve precise spatiotemporal delivery and produce lasting anti-tumor immune responses. Consequently, multidisciplinary collaboration is urgently warranted for the development and clinical application of tumor vaccines. First, oncologists will keep on exploring the molecular mechanisms of breast cancer occurrence and development to identify new antigens, and immunologists need to conduct in-depth studies regarding relevant mechanisms of tumor immunity, screen antigen epitopes with the best immunogenicity, and select the best vaccine formula (including linkers and adjuvants); then chemists and material scientists will assist in the design of vaccine programs, develop reliable vaccine platforms, and achieve effective delivery and targeted activation; in the preclinical research stage, pharmacologists are also needed to evaluate the safety and dynamics of the vaccine and find the best route for administration; finally and most importantly, clinicians are warranted to complete appropriate patient screening, drug monitoring, efficacy evaluation, and the discovery and application of evaluation markers. The concerted efforts of all participants will ultimately establish an efficient, safe, and accurate vaccine clinical application program, and promote tumor vaccines from ideal to reality.
